# DHX37 Is a Promising Prognostic Biomarker and a Therapeutic Target for Immunotherapy and Chemotherapy in HCC

**DOI:** 10.3390/cancers15215228

**Published:** 2023-10-31

**Authors:** Nanbin Liu, Hailong Zhang, Chunli Zhang, Zeyu Li, Limin Huang, Jin Sun, Junan Qi, Xi Deng, Na Huang, Yanhua Mu, Zongfang Li, Hongwei Tian

**Affiliations:** 1 National and Local Joint Engineering Research Center of Biodiagnosis and Biotherapy, The Second Affiliated Hospital of Xi’an Jiaotong University, Xi’an 710004, China; lnb509755573@stu.xjtu.edu.cn (N.L.); long753852@stu.xjtu.edu.cn (H.Z.); chunlizhang@xjtu.edu.cn (C.Z.); lzy2152@stu.xjtu.edu.cn (Z.L.); huanglimin@stu.xjtu.edu.cn (L.H.); jinsun2014@xjtu.edu.cn (J.S.); d605365632@stu.xjtu.edu.cn (X.D.); huangna123@xjtu.edu.cn (N.H.); muyanhua@xjtu.edu.cn (Y.M.); 2 Shaanxi Provincial Clinical Research Center for Hepatic & Splenic Diseases, Xi’an 710004, China; qijunan.06@stu.xjtu.edu.cn; 3 Tumor and Immunology Center of Precision Medicine Institute, Xi’an Jiaotong University, Xi’an 710049, China; 4 The First Ward of Hepatobiliary Pancreatic and Spleen Surgery, Baoji Municipal Central Hospital, Baoji 721008, China

**Keywords:** liver hepatocellular carcinoma, DHX37, prognosis, chemotherapy, immunotherapy

## Abstract

**Simple Summary:**

The DEAD/H-box RNA helicase family is widely involved in tumor progression. Previously, many studies have found that DHX9, DHX15, DDX24, DHX29, etc. are widely involved in tumor progression through various pathways, however, the role of DHX37, as an important member of the DEAD/H-box RNA helicase family, in hepatocellular carcinoma progression has been less studied. In this study, we analyzed the expression of DHX37 in hepatic hepatocellular carcinoma (HCC), its correlation with clinicopathological features, potential prognostic value, and the effect of DHX37 expression on chemotherapy and immunotherapy of hepatocellular carcinoma by using bioinformatics tools such as GDSC, HPA, STRING, TISCH, and TIMER2. Clinical HCC samples were used to validate DHX37 expression in HCC and the correlation between DHX37 expression and immune cell infiltration. Our study suggests that DHX37 is a novel prognostic marker for hepatocellular carcinoma and is valuable for predicting response to chemotherapy and immunotherapy.

**Abstract:**

DHX37, a member of the DEAD/H-box RNA helicase family, has been implicated in various diseases, including tumors. However, the biological characteristics and prognostic significance of DHX37 in HCC remain unclear. In this study, we use R software 3.6.3 and multiple bioinformatics analysis tools, such as GDSC, HPA, STRING, TISCH, and TIMER2, to analyze the characterization and function of DHX37 in HCC. In addition, Western blot (WB) and immunohistochemistry (IHC) based on clinical samples validated some of the findings. DHX37 was more highly expressed in HCC samples compared to adjacent non-tumor tissues. Higher DHX37 expression is correlated with various clinicopathological characteristics in HCC, including AFP, adjacent hepatic tissue inflammation, histologic grade, T stage, and pathologic stage. Survival analysis revealed that the high DHX37 group had significantly shorter overall survival (OS), progress-free interval (PFI), and disease-specific survival (DSS) compared to the low DHX37 group. By analyzing the correlation between DHX37 and the IC50 of chemotherapeutic drugs, the results showed that DHX37 expression level was negatively correlated with the IC50 of 11 chemotherapeutic drugs. Further analysis indicated that DHX37 and its co-expressed genes may play important roles in activating the cell cycle, DNA repair, chemokine signaling pathways, and regulating the immune response, which leads to a poor prognosis in HCC. High expression of DHX37 is an independent risk factor for poor prognosis in HCC, and DHX37 is expected to be a potential target to inhibit tumor progression. Targeting DHX37 may enhance chemotherapeutic drug sensitivity and immunotherapeutic efficacy in HCC.

## 1. Introduction

HCC is one of the most common digestive tumors and the third most frequent cause of cancer-associated mortality [1]. Various treatment modalities, including surgery, chemotherapy, radiotherapy, and immunotherapy, have been proven effective in managing HCC, but the prognosis of HCC remains dismal due to factors such as postoperative recurrence, treatment resistance, and difficulty in early diagnosis [2]. Previous studies have demonstrated the significance of many molecules in the occurrence, progression, therapeutic resistance, and immune evasion of HCC [3,4,5]. Mechanisms underlying HCC development have been extensively studied (although not fully understood); therefore, further exploration of novel biomarkers that can accurately and timely predict the biological behavior and prognosis of HCC is important for the prevention and treatment of HCC.

RNA helicases, which are widespread and highly conserved enzymes, have been extensively investigated due to their involvement in vital biological processes such as transcription, translation, and post-translational modifications. Recent studies have reported that RNA helicases play a role in the progression of a variety of tumor types, including DHX9 [6], DHX15 [7], DDX24 [8], DHX29 [9], and DHX36 [10]. Dong MB et al. demonstrated that the anti-tumor activity of DHX37 against breast cancer was markedly enhanced after knockdown of DHX37 in T-cells [11]. Zhen Liu et al. demonstrated that DHX37 can cooperate with PLRG1 to Drive Superenhancer-Mediated Transcription of Cyclin D1 and Facilitates Liver Cancer Progression. However, it is not clear whether DHX37 can promote HCC progression through other pathways [12], so it is necessary to further explore the role of DHX37 in HCC.

In order to understand the role of DHX37 in the development of HCC. In this study, we obtained HCC data from the TCGA database and the GEO database and thoroughly analyzed the value of DHX37 in HCC using R software and various bioinformatics analysis tools. Additionally, we performed essential experiments to validate the value and characteristics of DHX37 in clinical samples. Our study aims to elucidate the value of DHX37 as a prospective therapeutic target and prognostic indicator in HCC and to provide potential leads for the development of new drugs for the treatment of HCC.

## 2. Materials and Methods

### 2.1. Data Acquisition and Processing

RNAseq data in HTSeq-FPKM format, as well as gene expression, immune infiltration, and corresponding clinical information on 374 HCC patients and 50 normal liver tissue cases, were procured from the TCGA database (https://portal.gdc.cancer.gov/, accessed on 29 January 2023). We then converted the FPKM format RNAseq data to TPM format and subsequently conducted log2 transformations to facilitate further analysis [13]. 

Additionally, two HCC-related datasets, GSE45267 and GSE121248, were obtained from the GEO database (https://www.ncbi.nlm.nih.gov/geo/, accessed on 29 January 2023). Expression analysis and visualization were conducted through the “ggplot2” package in the R software.

The Human Protein Atlas (HPA) (https://www.proteinatlas.org/, accessed on 29 January 2023) [14] is a comprehensive public database of human protein information, containing protein data from cells, tissues, and organs that are primarily used to analyze the expression of specific proteins. We used HPA to investigate the expression of DHX37 in HCC tissue and immune cells.

The Tumor Immune Single-Cell Hub (TISCH) database (http://tisch.comp-genomics.org/home/, accessed on 29 January 2023) [15] includes single-cell transcriptome data for 15 types of tumor tissues and immune cell subtypes. Additionally, the database provides annotations on immune cell subtypes and signaling pathways, as well as various bioinformatic tools for analysis. We utilized the TISCH database to analyze the expression of DHX37 at the single-cell level.

### 2.2. Patients and Tissue Samples

Paraffin-embedded samples from 80 cases of HCC and 5 cases of normal liver tissue were subjected to immunohistochemical staining. Additionally, a collection of five pairs of frozen HCC tissues and corresponding non-tumor liver tissues was used for Western blot analysis. All tissues were obtained from the sample bank of the National and Local Joint Engineering Research Center of Biodiagnosis and Biotherapy at the Second Affiliated Hospital of Xi’an Jiaotong University.

Clinical data were gathered for all patients, including age, gender, BMI, preoperative AFP, bilirubin, lymph node metastasis, vascular invasion, TNM staging, and tumor differentiation. After surgery, all patients received regular follow-up, with imaging and laboratory tests conducted every 3–6 months. Patients were followed up until June 2023.

Informed consent was obtained from all patients, and this study was approved by the ethics committee of the Second Affiliated Hospital of Xi’an Jiaotong University.

### 2.3. Analysis of DHX37 Expression and with Clinicopathological Characteristics and Prognosis

The correlation between DHX37 expression and clinical pathological characteristics, including age, gender, BMI, AFP, bilirubin, tumor staging, grading, and vascular invasion, was analyzed in HCC tissues. The visualization was completed using the "ggplot2" package.

Overall survival (OS) is defined as the time from treatment to death, or progression. Free Interval (PFI), also known as Progression Free Survival (PFS), is the length of time during and after the treatment of a disease, such as cancer, that a patient lives with the disease but it does not get worse. disease Specific survival (DSS) is the percentage of people who died from a specific disease in a defined period of time. Patients who died from causes other than the disease being studied are not counted. OS, DSS, and PFI were used to evaluate the relationship between DHX37 expression and patient prognosis, and OS was used to evaluate the relationship between DHX37 expression and patient prognosis in different subgroups. Survival curves were plotted using the “survival” package. Receiver operating characteristic curve (ROC), time-dependent ROC, and risk factor analyses were conducted using the “pROC”, “timeROC”, and “ggrisk” packages, respectively, and the results were visualized using the “ggplot2” package. The correlation of DHX37 expression with clinicopathological characteristics and the prognosis was further investigated in our collected cases.

### 2.4. Analysis of DHX37 Expression and Chemotherapy Response

The GDSC database (Cancer Drug Sensitivity Genomics) (https://www.cancerrxgene.org/, accessed on 29 January 2023) [16] was utilized to predict the chemotherapy response of each sample. The R package “prophetic” was used to facilitate the prediction process, estimating the half-maximal inhibitory concentration IC50 of the samples using ridge regression. The default parameter values were used, and duplicate gene expression was summarized as the mean value.

### 2.5. Differentially Expressed Gene and Enrichment Analysis

Based on the median expression levels of DHX37 mRNA in the obtained expression data, patients were divided into high- and low-expression groups. The “ggplot2” package in R was used to analyze the differentially expressed genes between the high and low DHX37 expression groups. Genes with |log2(FC)| > 1.0 and a *p*-value < 0.05 were selected for analysis. Meanwhile, the top 30 genes with the strongest correlation with DHX37 in HCC were obtained and visualized.

The differentially expressed genes between DHX37 high and low expression groups were subjected to Gene ontology (GO) analysis and gene set enrichment analysis (GSEA) using the “clusterProfiler” package in R. We employed the C2.Cp.v7.2. symbols.gmt [Curated] as the reference gene set for KEGG pathways. Each enriched pathway in each phenotype was categorized using a normalized enrichment score (NES) and an adjusted *p*-value, and a gene set was considered significantly enriched when it met both a false discovery rate (FDR) < 0.25 and an adjusted *p* < 0.05.

### 2.6. Protein-Protein Interaction Network

The STRING database (https://cn.string-db.org/, accessed on 29 January 2023) [17], a publicly available online bioinformatics analysis tool, was employed to analyze the protein-protein interaction network of DHX37, from which we selected the top 30 genes with the highest interaction scores.

### 2.7. Correlation Analysis of DHX37-Related Genes in Pan-Cancer

The top 10 genes with the highest correlation in the co-expression network and the top 10 genes with the highest interaction scores in the PPI network were selected. The correlation of DHX37 with these genes in pan-cancer tissues was analyzed using TIMER2 (Tumor Immune Estimation Resource 2.0) (http://timer.cistrome.org/, accessed on 29 January 2023) [18].

### 2.8. Prognostic Value and Functional Analysis of DHX37-Related Genes

The prognostic value and functional analysis of DHX37-related genes were further analyzed using the "survival" package in R software for the top 10 genes in both networks. The connection between the most relevant genes of DHX37 and some tumor-related signaling pathways was finally analyzed using GSCALite (https://www.editorialmanager.com/jtrm/default1.aspx, accessed on 29 January 2023) [19].

### 2.9. Correlation Analysis of DHX37-Related Genesand TIME

We used the “GSVA” package in R software to assess the correlation between DHX37 and its related genes, and 24 immune cell signature genes were used to represent the immune infiltration of these genes [20]. In addition, we also evaluated the correlation between DHX37 and its related genes and chemokines, immunostimulators, and immunoinhibitors. All results were visualized as heat and scatter plots using the “ggplot2” package. Spearman correlation was used for statistical methods.

The correlation of DHX37 (biorbyt, Cambridge, UK) with CD68 (Affinity Biosciences, Cincinnati, OH, USA) and PDL1 (proteintech, Rosemont, IL, USA) was validated in 40 HCC samples using immunohistochemical staining.

### 2.10. Immunohistochemistry

The histological chips were first dewaxed sequentially with xylene I, xylene II, anhydrous ethanol, 95% ethanol, 90% ethanol, and 85% ethanol, followed by antigen repair using sodium citrate and cooling to room temperature. 3% hydrogen peroxide was used to inhibit endogenous peroxidase activity (20 min) and closed in goat serum (20 min). Then, DHX37 rabbit polyclonal antibody was diluted at a ratio of 1:200, dropped on slides, and incubated overnight at 4 °C. After removal from the refrigerator the next day, secondary antibodies were added dropwise (30 min) at a dilution ratio of 1:50, color development reactions were performed using diaminobenzidine (DAB) staining, hematoxylin staining was used, and the sections were dehydrated, transparent, and sealed with neutral gum.

We took five random photographs of each section under the microscope and performed semi-quantitative analysis using Image-Pro Plus 6.0 image analysis software. Measurements included the area and density of the stained area and integrated optical density (IOD), and the five IOD data from each section were averaged as a valid indicator for analysis and used to represent the expression level of DHX37, CD68, and PDL1.

### 2.11. Western Blotting

Firstly, protein samples were collected from tissues using RIPA lysate extraction, and protein concentrations were detected using the BCA protein kit. Protein samples were then separated on 10% SDS-PAGE gels and transferred to PVDF membranes at low temperature, closed with 5% skim milk for 2 h. PVDF membranes were placed in diluted primary antibody and incubated overnight at 4 °C (DHX37: 1:2000, proteintech, USA; GAPDH: 1:1000, ABclonal, Woburn, MA, USA). The following day, PVDF membranes were incubated with horseradish peroxidase-coupled secondary antibodies (1:2000) for 2 h. Finally, protein expression was assessed using the ECL kit and quantified in Image J software v1.8.0.

### 2.12. Statistical Analysis

For the data from TCGA, GEO, and our clinical sample, some packages were used in R (e.g., “GSVA” package, ”ggplot2” package, “pROC” package, “survival” package, etc.), and Graphpad Prism 8.0 was used for data processing and visualization. The statistical methods involved included the Wilcoxon rank-sum test, Welch one-way ANOVA, *t*-test, Bonferroni correction, Kruskal-Wallis, and Spearman correlation, which were mainly used to compare data differences between groups. Univariate Cox regression analysis and multivariate Cox regression were used to assess the prognostic value of DHX37 expression and clinicopathological features. The Kaplan-Meier analyses were applied to the OS, PFI, and DSS with the log-rank test. All results were statistically significant at *p* < 0.05 for differences.

## 3. Results

### 3.1. DHX37 Is Highly Expressed in HCC

**Figure 1:**
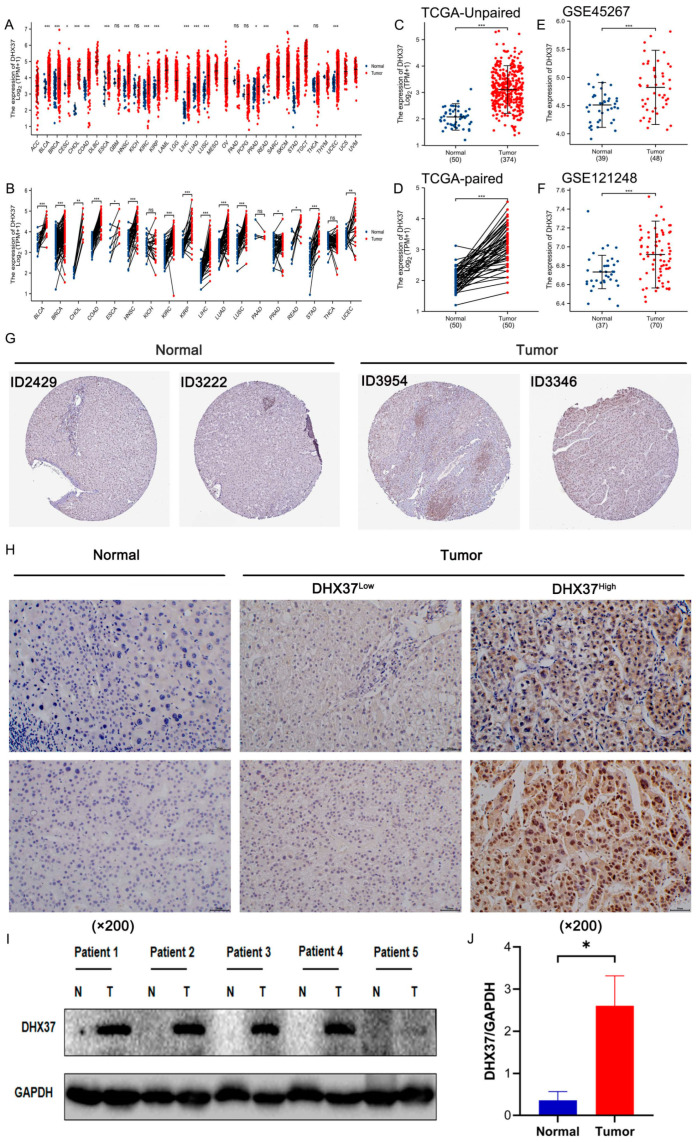
DHX37 expression landscape. (**A**,**B**) TCGA database of DHX37 expression levels in unpaired/paired pan-cancerous tissues. (**C**,**D**) TCGA database of DHX37 expression levels in unpaired/paired HCC. (**E**,**F**) Expression levels of DHX37 in HCC in the GEO database. (**G**,**H**) Typical DHX37 immunohistochemical staining of hepatocellular carcinoma and normal tissues from HPA and our clinical samples. (**I**,**J**) Western blot detection of DHX37 protein expression levels in specimens from 5 pairs of HCC patients (T: tumor, N: normal). (* *p* < 0.05; ** *p* < 0.01; *** *p* < 0.001). Newly added WB citation about Supplementary Materials in figure, please confirm. The densitometry readings/intensity ratio for normal tissue were 0.12, 0.47, 0.04, 0.02, and 1.14, respectively; and for tumor tissue, 2.05, 1.68, 4.68, 3.8, and 0.82, respectively.

High expression of genes in tumor tissues predicts that it may promote tumor progression. First, we found that DHX37 was significantly highly expressed in 16 unpaired tumor tissues and 15 paired tumor tissues by analyzing the pan-cancer data obtained from TCGA, including BLCA (Bladder urothelial carcinoma), BRCA (Breast invasive carcinoma), CESC (Cervical squamous cell carcinoma and endocervical adenocarcinoma) (high expression in unpaired samples, no difference in paired samples), CHOL (Cholangiocarcinoma), COAD (Colon adenocarcinoma) (Esophageal carcinoma), HNSC (Head and Neck squamous cell carcinoma), KIRC (Kidney renal clear cell carcinoma), KIRP (Kidney renal papillary cell carcinoma), HCC, LUAD (Lung adenocarcinoma), LUSC (Lung squamous cell carcinoma), PRAD (Prostate adenocarcinoma), READ (Rectum adenocarcinoma), STAD (Stomach adenocarcinoma), UCEC (Uterine Corpus Endometrial Carcinoma) (Figure 1A,B). In particular, TCGA-HCC data analysis revealed that DHX37 was significantly highly expressed in both paired and unpaired tumor tissues (Figure 1C,D). This finding was further supported by analyzing two GEO datasets (GEO number), which also showed DHX37 was significantly highly expressed in HCC compared to normal tissues (Figure 1E,F). In addition, our immunohistochemical images of DHX37 in HCC tissues and normal liver tissues obtained from HPA were consistent with the above findings (Figure 1G). To further validate these findings, we performed IHC and WB assays on the collected HCC tissues and paired normal tissues, and the results demonstrated that DHX37 was significantly highly expressed in HCC (Figure 1H–J).

### 3.2. Correlation between DHX37 Expression and Clinicopathological Characteristics

**Figure 2:**
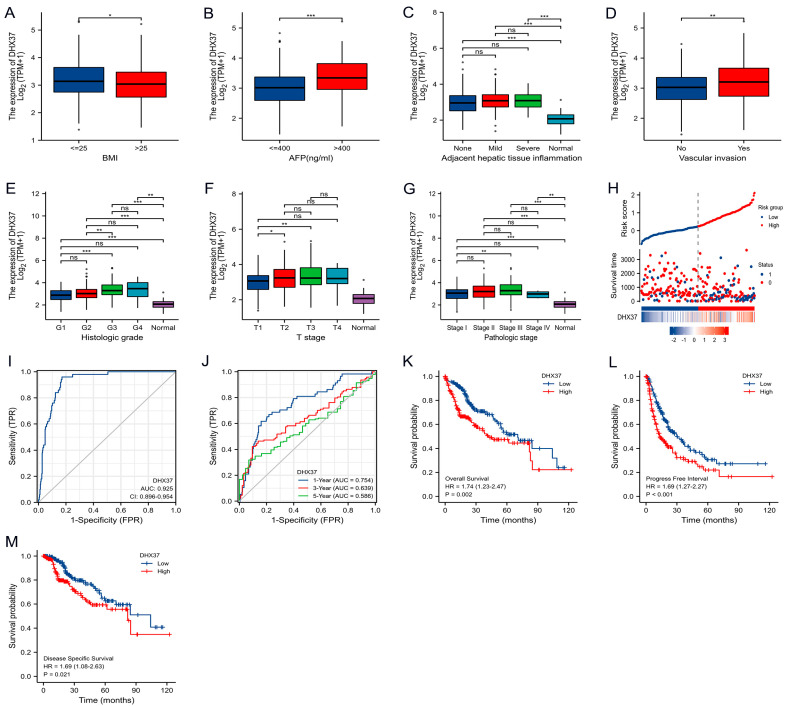
The correlation between DHX37 expression and clinicopathological features and the prognostic value of DHX37 in HCC. (**A**–**G**) Correlation of DHX37 expression with BMI, AFP, Adjacent hepatic tissue inflammation, Vascular invasion, Histologic grade, T stage, and Pathologic stage, data from the TCGA cohort. (**H**) DHX37 expression distribution and survival status. 0: dead; 1: alive. (**I**) ROC diagnostic curve of DHX37. (**J**) Time-dependent ROC curves of DHX37. (**K**–**M**) Prognostic value of DHX37 for OS, PFI, and DSS of HCC in the TCGA cohort. (* *p* < 0.05; ** *p* < 0.01; *** *p* < 0.001, ns means: no significance).

Based on TCGA data, we analyzed the correlation between DHX37 expression and clinical pathological features in 374 HCC patients. Results showed that DHX37 expression was associated with AFP (ng/mL) (*p* = 0.005), tumor status (*p* = 0.009), residual tumor (*p* = 0.017), vascular invasion (*p* = 0.035), and histologic grade (*p* = 0.019) (Table 1). High DHX37 expression was correlated with high BMI, AFP, adjacent hepatic tissue inflammation, vascular invasion, histologic grade, T stage, and pathologic stage (Figure 2A–G). However, there was no significant correlation with other clinical-pathological features (Figure S1A–E).

**Figure 3:**
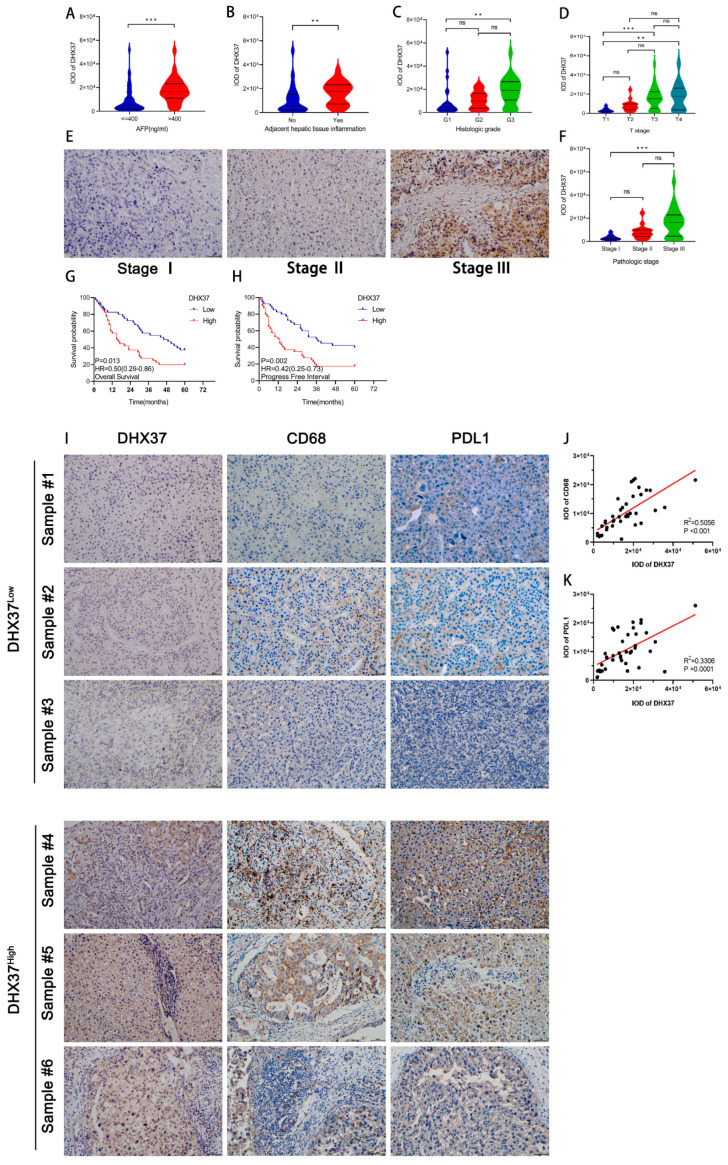
The prognostic value of DHX37 and the correlation between its expression level and that of CD68 and PDL1 were validated based on clinical samples. (**A**–**F**) Correlation of DHX37 expression with AFP, adjacent liver tissue inflammation, histological grade, T-stage, and pathological stage, data from clinical samples, and IHC images of DHX37 expression levels in different clinical stages. (**G**,**H**) Prognostic value of DHX37 for OS and PFI of HCC in clinical samples. (**I**–**K**) Correlation of DHX37 expression levels with CD68 and PDL1 expression levels (** *p* < 0.01; *** *p* < 0.001).

To further validate the above conclusions, we verified the correlation between clinical information and DHX37 expression in 80 HCC patients from our hospital. Results revealed that high DHX37 expression was correlated with high AFP, adjacent hepatic tissue inflammation, histologic grade, T stage, and pathologic stage (Figure 3A–F), but there was no significant correlation with other clinical-pathological features (Figure S1F–J).

### 3.3. Prognostic Value Analysis of DHX37

In the COX regression model, univariate Cox regression indicated that tumor status (*p* < 0.001), pathologic T stage (*p* < 0.001), pathologic M stage (*p* = 0.017), and DHX37 level (*p* < 0.001) were risk factors for the OS of HCC. Multivariate Cox regression showed that tumor status (*p* = 0.006), pathologic T stage (*p* < 0.001), and DHX37 level (*p* = 0.007) were independent risk factors for the OS of HCC (Table 2).

We used R software to create a DHX37 risk factor plot to illustrate the distribution of DHX37 expression and the survival status of HCC patients (Figure 2H). The blue dots in the figure represent the survival status, while the red dots represent the dead status. As the risk score increases, the number of red dots also increases, indicating that patients with high DHX37 expression have a higher risk of death.

Furthermore, ROC analysis indicated that high DHX37 expression may lead to a poor prognosis for HCC patients (AUC = 0.925, 95% CI: 0.896–0.954). Time-dependent ROC analysis revealed that DHX37 also had a high value in predicting the 1, 3, and 5-year OS of HCC patients (Figure 2I,J).

Using R software, we analyzed the OS, PFI, and DSS of HCC patients from TCGA. The results showed that high expression of DHX37 was associated with shorter OS (HR = 1.74, 95% CI: 1.23–2.47, *p* = 0.002), PFI (HR = 1.69, 95% CI: 1.27–2.27, *p* < 0.001), and DSS (HR = 1.69, 95% CI: 1.08–2.63, *p* = 0.021) (Figure 2K–M). Survival analysis of our independent cohort of 80 HCC samples validated the above conclusions for OS (HR = 0.50, 95% CI: 0.29–0.86, *p* = 0.013) and PFI (HR = 0.42, 95% CI: 0.25–0.73, *p* = 0.002) (Figure 3G,H).

**Figure 4:**
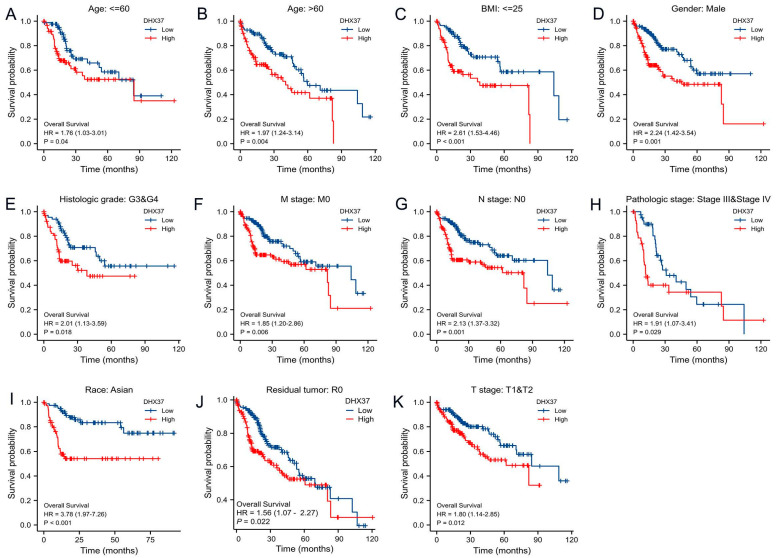
Kaplan-Meier curve analysis of the OS of DHX37 in different clinical subtypes. (**A**) Age ≤ 60, (**B**) Age > 60, (**C**) BMI ≤ 25, (**D**) Gender: male, (**E**) Histologic grade: G3 and G4 (**F**) M stage: M0, (**G**) N stage: N0, (**H**) Pathologic stage: Stage III and Stage IV (**I**) Race: Asian, (**J**) Residual tumor: R0, (**K**) T stage: T1 and T2.

We further analyzed the prognostic value of DHX37 in terms of different clinical-pathological characteristics. The results showed that DHX37 still had prognostic value in 11 subtypes, including Age ≤ 60 (HR = 1.76), Age > 60 (HR = 1.97), BMI ≤ 25 (HR = 2.61), Gender: male (HR = 2.24), Histologic grade: G3 and G4 (HR = 2.01), M stage: M0 (HR = 1.85), N stage: N0 (HR = 2.13), Pathologic stage: Stage III and Stage IV (HR = 1.91), Race: Asian (HR = 3.78), Residual tumor: R0 (HR = 1.56), T stage: T1 and T2 (HR = 1.80) (Figure 4). However, it did not have prognostic value in the other 20 subtypes (Figure S2). These findings suggest that DHX37 expression holds prognostic value in HCC and may serve as a potential biomarker for predicting patient outcomes.

### 3.4. Differentially Expressed Gene and Enrichment Analysis

**Figure 5:**
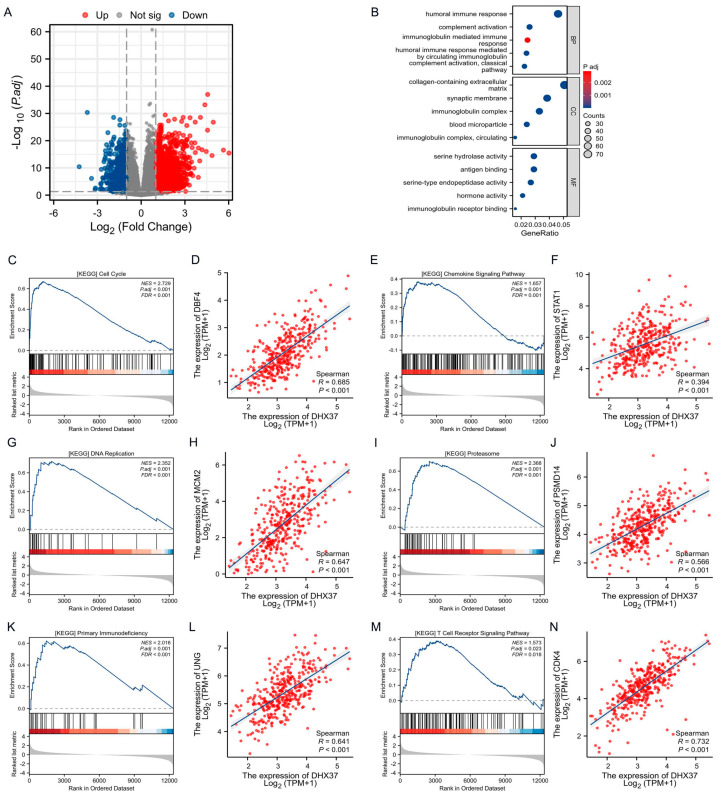
Differentially expressed gene (DEG) analysis and enrichment analysis of DHX37. (**A**) Volcano map of DEGs. (**B**) GO analysis of DHX37 differentially expressed genes. (**C**–**N**) GSEA analysis between the DHX37 low expression group and the DHX37 high expression group, and correlation analysis of DHX37 with core genes in each pathway.

A total of 2919 DEGs (of which 838 genes were down-regulated and 2081 genes were up-regulated) were clarified after single-gene differential analysis of DHX37-related genes using R software (Figure 5A). To understand the functions of these genes, GO functional analysis of DHX37-related DEGs was performed, and the results showed a major effect on humoral immune response, complement activation, and immunoglobulin-mediated immune response. The results showed that the functional analysis of DHX37-related DEGs focused mainly on humoral immune response, complement activation, immunoglobulin-mediated immune response, humoral immune response mediated by circulating immunoglobulin, complement activation classical pathway, collagen-containing extracellular matrix, synaptic membrane, immunoglobulin complex, blood microparticle, immunoglobulin complex circulating, serine hydrolase activity, antigen binding, serine-type endopeptidase activity, hormone activity, and immunoglobulin receptor binding were regulated (Figure 5B).

Next, to identify the DHX37-associated KEGG pathway, we also performed a GSEA analysis. The results showed that high DHX37 was significantly associated with some signaling pathways, for example, Cell Cycle (NES = 2.729, P.adj < 0.001), Chemokine Signaling Pathway (NES = 1.657, P.adj = 0.001), DNA Replication (NES = 2.352, P.adj < 0.001), Proteasome (NES = 2.368, P.adj < 0.001), Primary Immunodeficiency (NES = 2.016, P.adj = 0.001), T Cell Receptor Signaling Pathway (NES = 1.573, P.adj = 0.023). In addition, correlation analysis showed that the core molecules of the above six pathways and the expression of DHX37 were significantly correlated (Figure 5C–N). These results suggest that the mechanism of DHX37 regulation of downstream pathways may be through targeting these molecules, providing new targets for subsequent anticancer drug development.

### 3.5. Identification of DHX37-Related Genes

**Figure 6:**
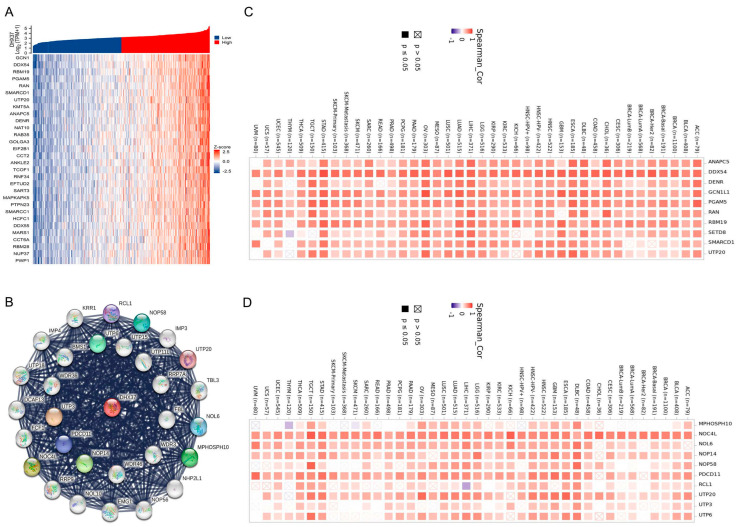
DHX37-related gene identification. (**A**) The top 30 genes with the highest correlation in the co-expression network. (**B**) PPI analysis of the top 30 genes with the highest co-interaction scores. (**C**) Heat map of the top 10 correlations in the co-expression network in pan-cancer. (**D**) Heat map of the top 10 correlations in the PPI network in pan-cancer.

We performed an analysis to identify a DHX37-related gene using RNAseq data obtained from the TCGA database. In R software, we analyzed the correlation between DHX37 and other genes; the top 30 genes with the highest correlation were visualized as a heat map (Figure 6A). To investigate protein-protein interactions (PPI) involving DHX37, we utilized the STRING tool and visualized the top 30 proteins with the highest scores (Figure 6B).

The top 10 genes from the correlation analysis results and the top 10 genes from the PPI were selected, and the co-expression of these genes with DHX37 in pan-cancer was analyzed by TIMER2, which showed that these 20 genes were co-expressed with DHX37 in most cancers (Figure 6C,D). These findings provide insights into the potential interactions and co-expression patterns of DHX37-related genes in various cancer types.

### 3.6. Prognostic Value and Biological Function Analysis of DHX37-Related Genes

**Figure 7:**
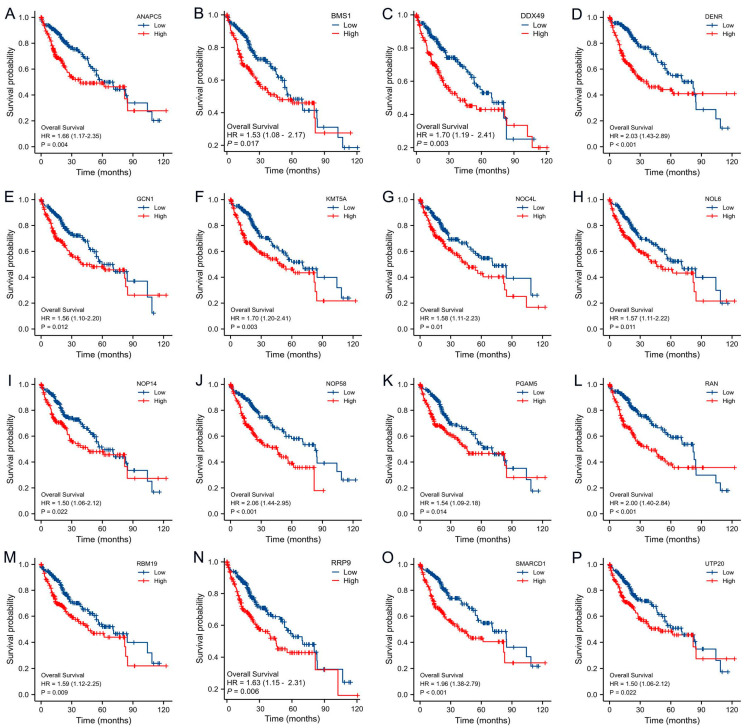
Survival analysis of DHX37-related genes. (**A**) ANAPC5, (**B**) BMS1, (**C**) DDX49, (**D**) DENR, (**E**) GCN1, (**F**) KMT5A, (**G**) NOC4L, (**H**) NOL6, (**I**) NOP14, (**J**) NOP58, (**K**) PGAM5, (**L**) RAN, (**M**) RBM19, (**N**) RRP9, (**O**) SMARCD1, (**P**) UTP20.

Kaplan–Meier survival curve analyses of the top 10 genes with the highest correlation in the co-expression network and the top 10 genes with the highest interaction score in the PPI network showed that 16 genes in HCC are associated with a poor prognosis. ANAPC5 (HR = 1.66, 95% CI:1.17–2.35, *p* = 0.004), BMS1 (HR = 1.53, 95% CI:1.08–2.17, *p* = 0.017), DDX49 (HR = 1.70, 95% CI:1.19–2.41, *p* = 0.003), DENR (HR = 2.03, 95% CI:1.43–2.89, *p* < 0.001), GCN1 (HR = 1.56, 95% CI. 1.10–2.20, *p* = 0.012), KMT5A (HR = 1.70, 95% CI:1.20–2.41, *p* = 0.003), NOC4L (HR = 1.58, 95% CI:1.11–2.23, *p* = 0.01), NOL6 (HR = 1.57, 95% CI:1.11–2.22, *p* = 0.011), NOP14 (HR = 1.50, 95% CI:1.06–2.12, *p* = 0.022), NOP58 (HR = 2.06, 95% CI:1.44–2.95, *p* < 0.001), PGAM5 (HR = 1.54, 95% CI:1.09–2.18, *p* = 0.014). RAN (HR = 2.00, 95% CI:1.40–2.84, *p* < 0.001), RBM19 (HR = 1.59, 95% CI:1.12–2.25, *p* = 0.009), RRP9 (HR = 1.63, 95% CI:1.15–2.31, *p* = 0.006), SMARCD1 (HR = 1.96, 95% CI:1.38–2.79, *p* < 0.001), UTP20 (HR = 1.50, 95% CI:1.06–2.12, *p* = 0.022) (Figure 7), while DDX54, MPHOSPH10, and UTP3 were not associated with prognosis in HCC (Figure S3A–C).

**Figure 8:**
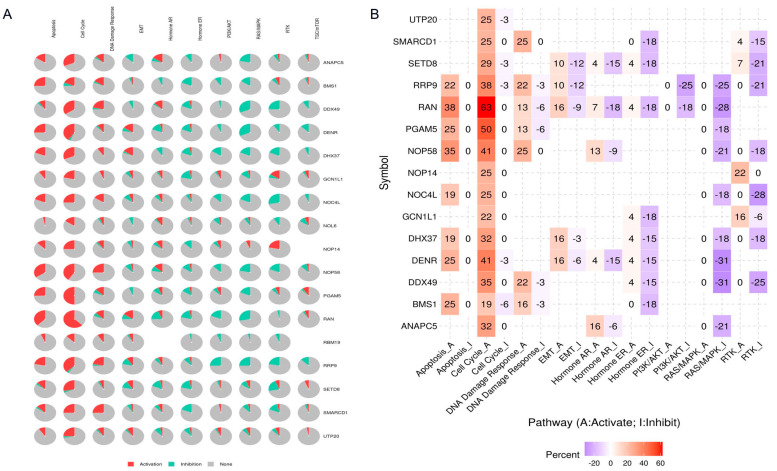
Common functional analysis of 17 genes in HCC. (**A**,**B**) Signaling pathway analysis of 17 genes.

We analyzed the common functions of DHX37 and the above 16 genes in HCC using the GSCALite tool, and the results suggest that the possible mechanism by which these genes promote HCC progression is through the regulation of the cell cycle and apoptosis (Figure 8).

### 3.7. Correlation between DHX37 Expression and Chemotherapy Response

**Figure 9:**
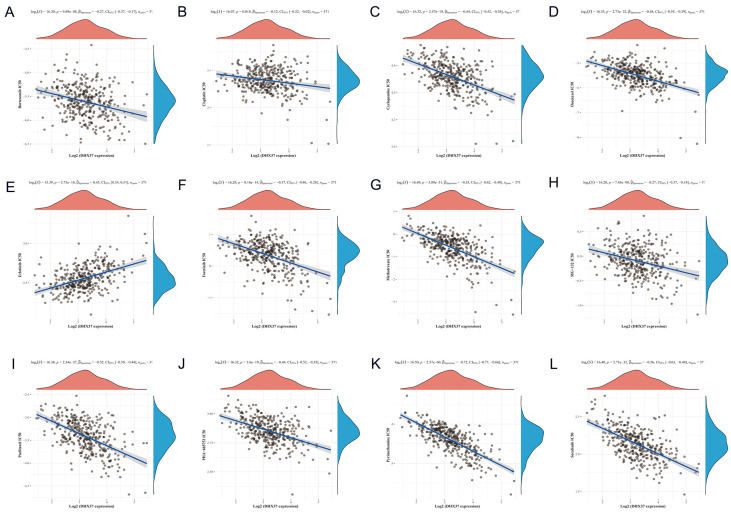
Correlation of DHX37 expression with chemotherapeutic drug half-inhibitory concentration (IC50). (**A**) Bortezomib, (**B**) Cisplatin, (**C**) Cyclopamine, (**D**) Docetaxel, (**E**) Erlotinib, (**F**) Foretinib, (**G**) Methotrexate, (**H**) MG-132, (**I**) Paclitaxel, (**J**) PHA-665752, (**K**) Pyrimethamine, and (**L**) Sorafenib.

We analyzed the correlation between DHX37 expression and chemotherapy response using data from GDSC. Our results showed that the expression of DHX37 was positively correlated with the IC50 of Erlotinib (Cor = 0.43, *p* < 0.001) and negatively correlated with the IC50 of Bortezomib (Cor = −0.27, *p* < 0.001), Cisplatin (Cor = −0.12, *p* = 0.018), Cyclopamine (Cor = −0.44, *p* < 0.001), Docetaxel (Cor = −0.48, *p* < 0.001), Foretinib (Cor = −0.37, *p* < 0.001), Methotrexate (Cor = −0.55, *p* < 0.001), MG-132 (Cor = −0.27, *p* < 0.001), Paclitaxel (Cor = −0.52, *p* < 0.001), PHA-665752 (Cor = −0.44, *p* < 0.001), Pyrimethamine (Cor = −0.72, *p* < 0.001), Sorafenib (Cor = −0.56, *p* < 0.001) (Figure 9). However, we did not observe a significant correlation between DHX37 and the IC50 of Dabrafenib, Mitomycin C, and Rapamycin (Figure S3D–F). These findings suggest that the expression level of DHX37 may have implications for personalized therapeutic drug selection for individual patients. Further research is needed to validate these findings and explore the underlying mechanisms behind these correlations.

### 3.8. DHX37 Expression Level in Immune Cells

**Figure 10:**
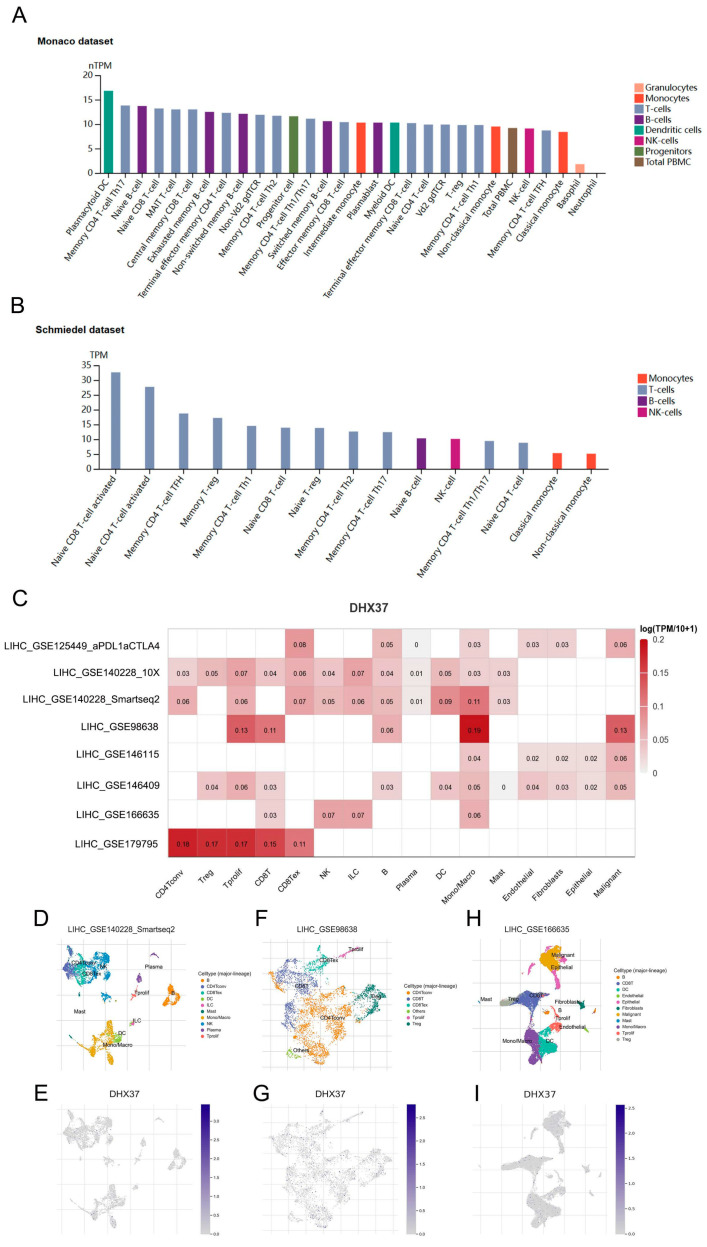
Expression level of DHX37 in immune cells. (**A**,**B**) DHX37 expression levels in various immune cells, data from the Monaco dataset and the Schmiedel dataset. (**C**) Expression levels of DHX37 in immune cells at the single cell level in eight HCC datasets, data from the TISCH database. (**D**–**I**) A single-cell-level global view of all tissues and DHX37 expression sites is shown in three HCC datasets.

We analyzed the expression levels of DHX37 in different immune cells using the HPA and TISCH databases. In the Monaco dataset, DHX37 was expressed in various immune cells, with the top five being Plasmacytoid DC, Memory CD4 T-cell Th17, Naive B-cell, Naive CD8 T-cell, and MAIT T-cell (Figure 10A). In the Schmiedel dataset, the top 5 immune cells with the highest expression levels of Dhx37 were Naive CD8 T-cell activated, Naive CD4 T-cell activated, Memory CD4 T-cell TFH, Memory T-reg, and Memory CD4 T-cell Th1 (Figure 10B). We used the TISCH database to analyze the expression of DHX37 at the single-cell level, and the results showed that DHX37 was mainly expressed in CD4 Tconv cells, Treg cells, Tprolif cells, CD8 T cells, and monocytes/macrophages in HCC (Figure 10C). Figure 10D,F,H shows the overall view at the single-cell level of the immune microenvironment of HCC, whereas Figure 10E,G,I shows the DHX37 expression sites in multiple immune cells.

### 3.9. Correlation Analysis of DHX 37-Related Genes and TIME

Immunity plays a significant role in tumor progression, and immunotherapy is an important research direction in current cancer treatment. The effect of immunotherapy mainly depends on TIME. Therefore, exploring the correlation of DHX37 and its related genes with the various components of TIME is necessary.

**Figure 11:**
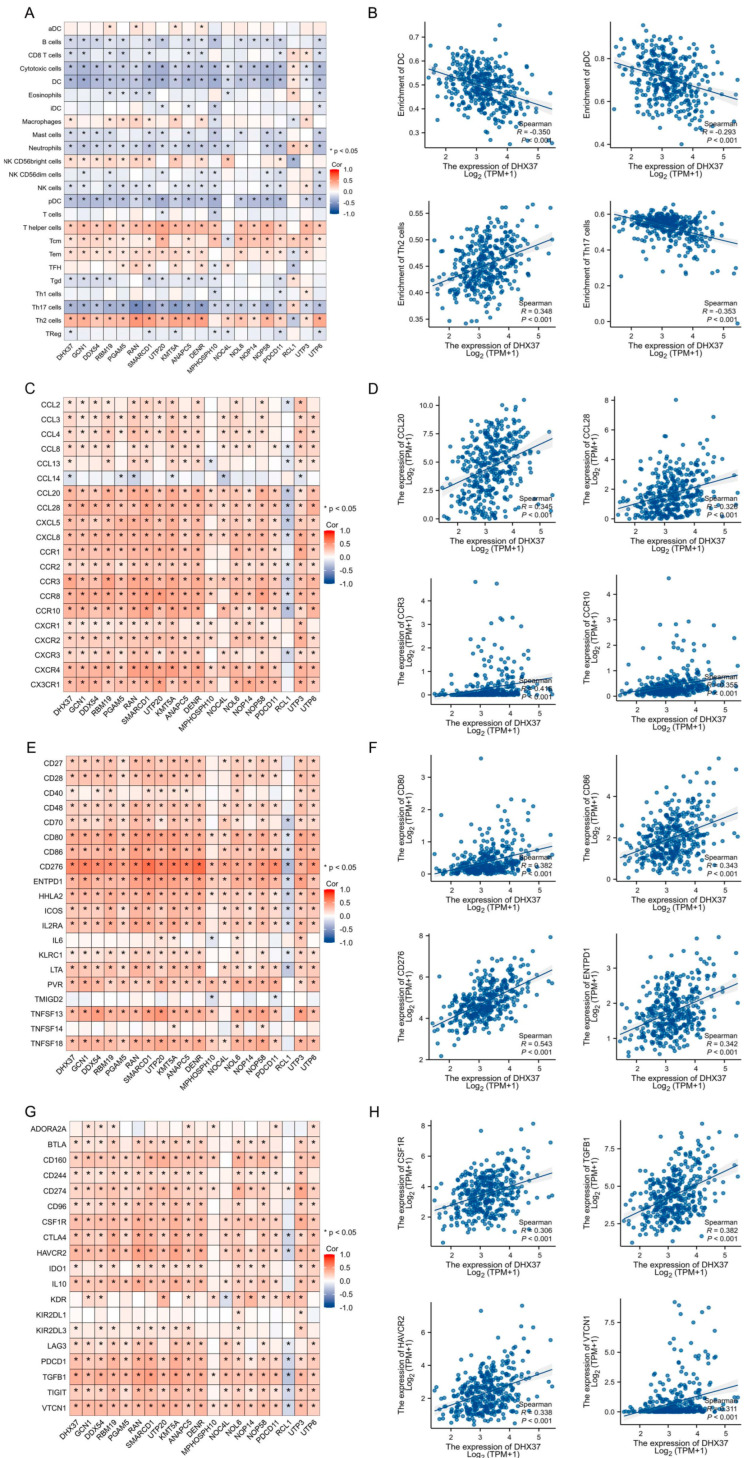
Correlation analysis of DHX37-related genes with TIME. (**A**) Heat map of the correlation between DHX37-related genes and 24 immune cell infiltrates. (**B**) The top 4 have the highest correlation between DHX37 and immune cell infiltration. (**C**) Heat map of the correlation between DHX37-related genes and chemokines. (**D**) The top 4 have the highest correlation between DHX37 and chemokines. (**E**) Heat map of the correlation between DHX37-related genes and immunostimulators. (**F**) The top 4 have the highest correlation between DHX37 and immunostimulators. (**G**) Heatmap of correlation between DHX37-related genes and immuneinhibitors. (**H**) The top 4 have the highest correlation between DHX37 and immunoinhibitors (* *p* < 0.05).

We analyzed the expression levels of DHX37 and its related genes in HCC concerning 24 immune cell infiltrations, chemokines, immune stimulators, and immune inhibitors using R software. All results were displayed in the form of heat maps, and the main results were presented in the form of scatter plots. The results showed that DHX37 and its related genes were negatively correlated with most immune cell infiltrations but positively correlated with most chemokines, immune stimulators, and immune inhibitors (Figure 11A,C,E,G). For example, DHX37 was negatively associated with DC (Cor = −0.350, *p* < 0.001), pDC (Cor = −0.293, *p* < 0.001), and Th17 cells (Cor = −0.353, *p* < 0.001), but positively associated with Th2 cells (Cor = 0.348, *p* < 0.001), CCL20 (Cor = 0.345, *p* < 0.001), CCL28 (Cor = 0.326, *p* < 0.001), CCR3 (Cor = 0.416, *p* < 0.001), CCR10 (Cor = 0.355, *p* < 0.001), CD80 (Cor = 0.382, *p* < 0.001), CD86 (Cor = 0.343, *p* < 0.001), CD276 (Cor = 0.543, *p* < 0.001), ENTPD1 (Cor = 0.342, *p* < 0.001), CSF1R (Cor = 0.306, *p* < 0.001), TGFB1 (Cor = 0.382, *p* < 0.001), HAVCR2 (Cor = 0.338, *p* < 0.001), and VTCN1 (Cor = 0.311, *p* < 0.001) (Figure 11B,D,F,H).

In addition, the IHC of clinical samples showed that CD68 and PDL1 expression was also relatively high in samples with relatively high DHX37 expression (Sample #4, Sample #5, and Sample #6). Moreover, correlation analysis showed that the expression level of DHX37 was positively correlated with the expression levels of CD68 and PDL1. (Figure 3I–K).

## 4. Discussion

DHX37, which is situated on human chromosome 12q24.31, consists of 1157 amino acids and comprises four functional domains: namely the helicase ATP-binding domain (RecA1), the helicase superfamily C-terminal domain (RecA2), the helicase-associated domain (HA2), and the oligonucleotide/oligosaccharide-binding fold domain (OB) [21]. The RNA helicase DHX37 participates primarily in RNA-related biological processes such as transcription, splicing, ribosome biogenesis, translation, and degradation [22,23]. Numerous studies have exhibited that DHX37 is linked to developmental disorders, neuro-related diseases, and other disorders [24,25]. HCC is a common malignant tumor; however, the relationship between DHX37 and HCC has not been comprehensively reported. In this study, we first analyzed the role of DHX37 in HCC by raw letter and further verified some of the conclusions by WB and IHC based on 85 clinical samples.

First, we found that DHX37 was highly expressed in most tumor tissues, including HCC. To further validate the above findings, we performed IHC and WB experiments based on clinical samples from HCC. The IHC results showed that DHX37 was highly expressed in HCC tissues compared to normal liver tissues and was mainly expressed in the nucleus. Second, we collected five pairs of HCC and adjacent non-tumor tissues for WB, and the results showed that DHX37 was significantly overexpressed in the tumor tissues of the four cases, except in one case where there was no significant difference in the expression of DHX37 between the tumor tissues and the adjacent non-tumor tissues. We speculate that this difference may be due to the heterogeneity of the samples and the individual differences of the tumors, so it is necessary to expand the sample size to further verify the expression of DHX37 in HCC.

It is commonly believed that HCC is a tumor highly associated with inflammation, and some factors in the inflammatory response may reduce immune activity and promote tumor progression through different pathways [26,27]. In addition, high AFP levels and vascular invasion are risk factors for the poor prognosis of HCC [28]. Analyses of TCGA and clinical samples showed that the DHX37 expression level was positively correlated with the AFP expression level and inflammatory infiltration around the tumor. This suggests that DHX37 may be involved in the genesis and progression of HCC, and its mechanism of promoting HCC progression may be related to the regulation of inflammatory responses and immune activity. Secondly, although there was no statistical difference between DHX37 expression and vascular invasion in our clinical samples, the TCGA data analysis table showed a positive correlation between DHX37 expression and vascular invasion, and we speculate that the reason may be the small sample size, and further validation of the sample size for the correlation between vascular invasion and DHX37 expression will be the focus of future studies.

Tumor prognostic assessment is one of the main functions of biological markers. We verified the prognostic value of DHX37 for HCC based on TCGA data and clinical samples. The results showed that OS, PFI, and DSS were significantly shorter in HCC patients with high expression of DHX37. DHX37 was accurate in predicting OS in HCC patients. In different clinical subtypes, DHX37 still had strong prognostic potential. These results suggest that DHX37 may be a valuable prognostic biomarker for HCC.

Chemotherapy is one of the adjuvant treatments for HCC, but the efficacy of chemotherapy varies widely between individuals. We analyzed the correlation between DHX37 expression levels and the IC50 of 15 chemotherapy drugs, and the results showed that DHX37 expression levels were negatively correlated with the IC50 of 11 chemotherapy drugs. These results indicate the potential of DHX37 to guide the personalized treatment of HCC patients.

Further analysis revealed that the following pathways were activated in samples with high DHX37 expression: cell cycle, chemokine signaling pathway, DNA replication, proteasome, primary immunodeficiency, and T cell receptor signaling pathway. Correlation analysis showed that DBF4, STAT1, MCM2, PSMD14, UNG, and CDK4 were core proteins of the above pathways, and correlation analysis indicated that all of them were positively correlated with DHX37 [29,30,31,32,33,34]. We hypothesized that DHX37 may promote HCC progression by interacting with these proteins and thus regulating the related pathways.

HCC is a highly immunosuppressive tumor, and immune cells, chemokines, stimulating factors, and suppressor factors in TIME are closely related to the occurrence and progression of HCC [35,36]. Correlation analysis of DHX37 and its related genes with TIME showed that DHX37 and its related genes were negatively correlated with the cellular infiltration of cells such as DC cells and CD8 T cells and positively correlated with the expression of proteins such as CCL20, CCR10, CD80, CD276, TGF-β, and VTCN1 protein expression. GO and KEGG analyses indicated that DHX37 may be involved in multiple immune activities, and we hypothesized that DHX37 may contribute to the progression of HCC by regulating the TIME of HCC.

Immunotherapy represented by PD1 inhibitors has achieved good efficacy but still suffers from a low response rate in tumor patients. We observed in our clinical samples that high DHX37 expression may lead to increased macrophage infiltration and up-regulation of PDL1 expression, which may be the reason for the poor immunotherapy outcome in patients with high DHX37 expression.

We also analyzed the expression of DHX37 in immune cells and showed that DHX37 was mainly present in plasma cell-like DCs, Naive CD8 T cells, activated Naive CD4 T cells, Treg cells, and monocytes/macrophages, which guided our subsequent study of the mechanism by which DHX37 regulates tumor immunity.

In this study, we conducted a preliminary investigation of the role of DHX37 in HCC by combining publicly available databases, clinical samples, and experimental evidence. The results suggest that the high expression of DHX37 in HCC may promote tumor progression, which is an important factor affecting tumor prognosis, and is potentially instructive in personalized chemotherapy and immunotherapy for HCC. Although some meaningful findings have been made, there are still some limitations in our study. For example, only 80 HCC samples were collected, and relevant functional experiments have not yet been carried out. Further research incorporating additional evidence and conducting in-depth studies will be necessary to validate and expand upon these findings.

## Figures and Tables

**Table 1:** Relationship between DHX37 expression and clinicopathological features in TCGA-HCC.

Characteristic	Low Expression of DHX37	High Expression of DHX37	*p*
n	187	187	
Age, n (%)			0.797
≤60	87 (23.3%)	90 (24.1%)	
>60	100 (26.8%)	96 (25.7%)	
Gender, n (%)			0.269
Female	66 (17.6%)	55 (14.7%)	
Male	121 (32.4%)	132 (35.3%)	
BMI, n (%)			0.409
≤25	85 (25.2%)	92 (27.3%)	
>25	85 (25.2%)	75 (22.3%)	
AFP(ng/mL), n (%)			0.005
≤400	121 (43.2%)	94 (33.6%)	
>400	23 (8.2%)	42 (15%)	
Albumin(g/dl), n (%)			0.497
<3.5	40 (13.3%)	29 (9.7%)	
≥3.5	121 (40.3%)	110 (36.7%)	
Prothrombin time, n (%)			0.585
≤4	108 (36.4%)	100 (33.7%)	
>4	50 (16.8%)	39 (13.1%)	
Adjacent hepatic tissue inflammation, n (%)			0.584
None	69 (29.1%)	49 (20.7%)	
Mild	52 (21.9%)	49 (20.7%)	
Severe	10 (4.2%)	8 (3.4%)	
Child-Pugh grade, n (%)			0.811
A	119 (49.4%)	100 (41.5%)	
B	10 (4.1%)	11 (4.6%)	
C	1 (0.4%)	0 (0%)	
Fibrosis ishak score, n (%)			0.633
0	42 (19.5%)	33 (15.3%)	
1/2	18 (8.4%)	13 (6%)	
3/4	13 (6%)	15 (7%)	
5/6	49 (22.8%)	32 (14.9%)	
Tumor status, n (%)			0.009
Tumor free	114 (32.1%)	88 (24.8%)	
With tumor	64 (18%)	89 (25.1%)	
Residual tumor, n (%)			0.017
R0	173 (50.1%)	154 (44.6%)	
R1	4 (1.2%)	13 (3.8%)	
R2	0 (0%)	1 (0.3%)	
Vascular invasion, n (%)			0.035
No	118 (37.1%)	90 (28.3%)	
Yes	48 (15.1%)	62 (19.5%)	
T stage, n (%)			0.267
T1	100 (27%)	83 (22.4%)	
T2	44 (11.9%)	51 (13.7%)	
T3	34 (9.2%)	46 (12.4%)	
T4	6 (1.6%)	7 (1.9%)	
N stage, n (%)			0.623
N0	123 (47.7%)	131 (50.8%)	
N1	1 (0.4%)	3 (1.2%)	
M stage, n (%)			1.000
M0	131 (48.2%)	137 (50.4%)	
M1	2 (0.7%)	2 (0.7%)	
Pathologic stage, n (%)			0.233
Stage I	94 (26.9%)	79 (22.6%)	
Stage II	43 (12.3%)	44 (12.6%)	
Stage III	35 (10%)	50 (14.3%)	
Stage IV	3 (0.9%)	2 (0.6%)	
Histologic grade, n (%)			0.019
G1	33 (8.9%)	22 (6%)	
G2	98 (26.6%)	80 (21.7%)	
G3	50 (13.6%)	74 (20.1%)	
G4	4 (1.1%)	8 (2.2%)	
OS event, n (%)			0.103
Alive	130 (34.8%)	114 (30.5%)	
Dead	57 (15.2%)	73 (19.5%)	
PFI event, n (%)			0.214
Alive	102 (27.3%)	89 (23.8%)	
Dead	85 (22.7%)	98 (26.2%)	
DSS event, n (%)			0.414
Alive	148 (40.4%)	139 (38%)	
Dead	36 (9.8%)	43 (11.7%)	

**Table 2:** Univariate and multivariate Cox regression analyses of Clinicopathological features associated with OS in HCC.

Characteristics	Total (N)	Univariate Analysis		Multivariate Analysis	
		Hazard Ratio (95% CI)	*p* Value	Hazard Ratio (95% CI)	*p* Value
Age	373				
≤60	177	Reference			
>60	196	1.205 (0.850–1.708)	0.295		
Gender	373				
Female	121	Reference			
Male	252	0.793 (0.557–1.130)	0.200		
BMI	336				
≤25	177	Reference			
>25	159	0.798 (0.550–1.158)	0.235		
Tumor status	354				
Tumor free	202	Reference		Reference	
With tumor	152	2.317 (1.590–3.376)	<0.001	1.917 (1.204–3.054)	0.006
AFP(ng/mL)	279				
≤400	215	Reference			
>400	64	1.075 (0.658–1.759)	0.772		
Albumin(g/dl)	299				
<3.5	69	Reference			
≥3.5	230	0.897 (0.549–1.464)	0.662		
Prothrombin time	296				
≤4	207	Reference			
>4	89	1.335 (0.881–2.023)	0.174		
Pathologic T stage	370				
T1&T2	277	Reference		Reference	
T3&T4	93	2.598 (1.826–3.697)	<0.001	2.194 (1.387–3.472)	<0.001
Pathologic N stage	258				
N0	254	Reference			
N1	4	2.029 (0.497–8.281)	0.324		
Pathologic M stage	272				
M0	268	Reference		Reference	
M1	4	4.077 (1.281–12.973)	0.017	1.686 (0.394–7.223)	0.481
Histologic grade	368				
G1&G2	233	Reference			
G3&G4	135	1.091 (0.761–1.564)	0.636		
Vascular invasion	317				
No	208	Reference			
Yes	109	1.344 (0.887–2.035)	0.163		
DHX37	373	1.782 (1.364–2.327)	<0.001	1.589 (1.135–2.224)	0.007

## Data Availability

Informed consent was obtained from all subjects involved in the study. The bioinformatics datasets used in this study are available at the corresponding websites, and raw data used during experiments will be made available by the authors upon reasonable request.

## Supplementary Materials

The following supporting information can be downloaded at: https://www.mdpi.com/article/10.3390/cancers15215228/s1, Figure S1. Correlation of DHX37 expression and clinicopathological characteristics. (A–E) Correlation of DHX37 expression with Age, Gender, Albumin, N stage, and M stage data from the TCGA cohort. (G–L) Correlation of DHX37 expression with Age, Gender, BMI, Albumin, and Vascular invasion. (* *p* < 0.05; ** *p* < 0.01; *** *p* < 0.001). Figure S2. OS of DHX37 in different clinical subtypes. Figure S3. Chemotherapeutic drug sensitivity analysis and survival analysis. (A–C) Correlation of DHX37 expression with the half-inhibitory concentration (IC50) of three chemotherapeutic drugs. (D–F) Survival analysis of DHX37-associated genes.
