# Supplementary material for: DHX37 Is a Promising Prognostic Biomarker and a Therapeutic Target for Immunotherapy and Chemotherapy in HCC
Source: Cancers (Basel). 2023 Oct 31;15(21):5228. doi: 10.3390/cancers15215228 (PMC10648173; doi:10.3390/cancers15215228)
Supplement: Supplementary file 1 [file cancers-15-05228-s001.zip › cancers-2644859-supplementary.pdf]

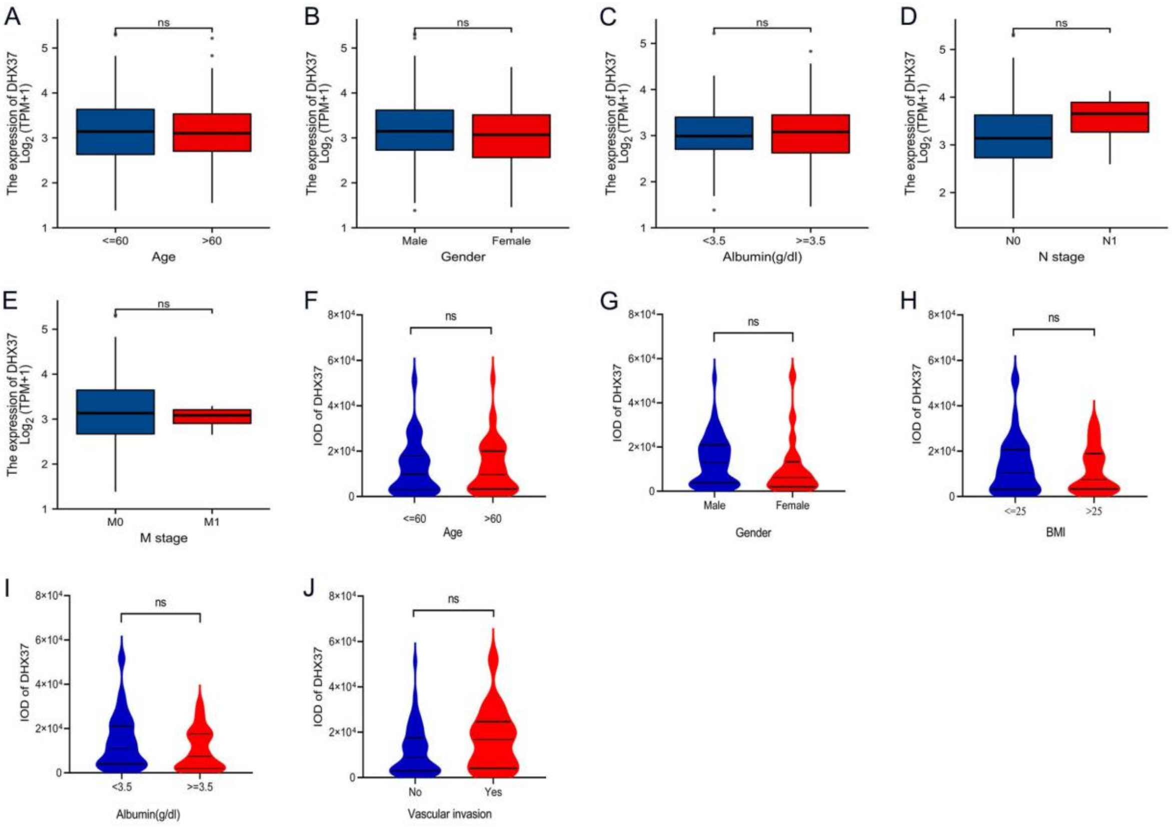

Fig. S1 Correlation of DHX37 expression and clinicopathological characteristics. (A-E) Correlation of DHX37 expression with Age, Gender, Albumin, N stage, M stage, data from TCGA cohort. (G-L) Correlation of DHX37 expression with Age, Gender, BMI, Albumin, and Vascular invasion. (*p<0.05; **p<0.01; ***p<0.001);

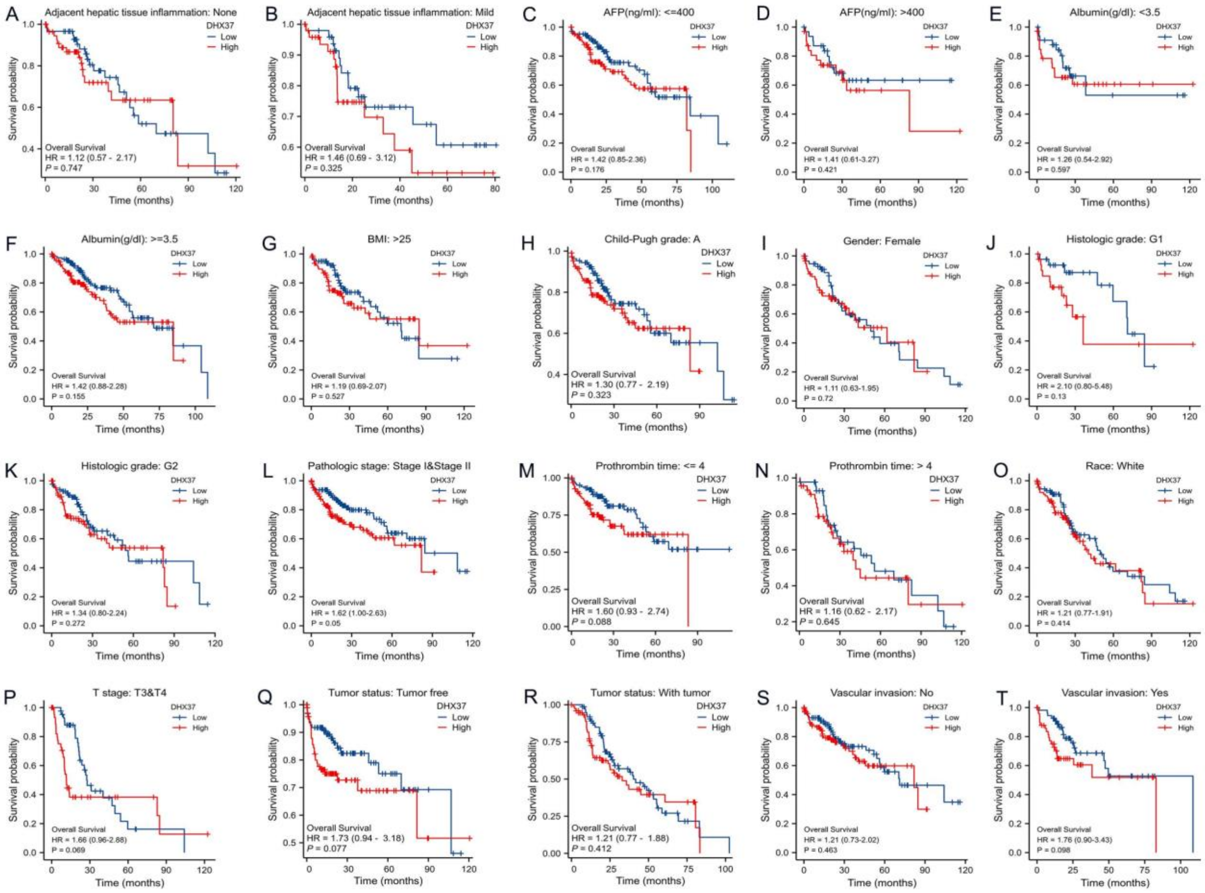

Additional file S1: Fig. S2 OS of DHX37 in different clinical subtypes

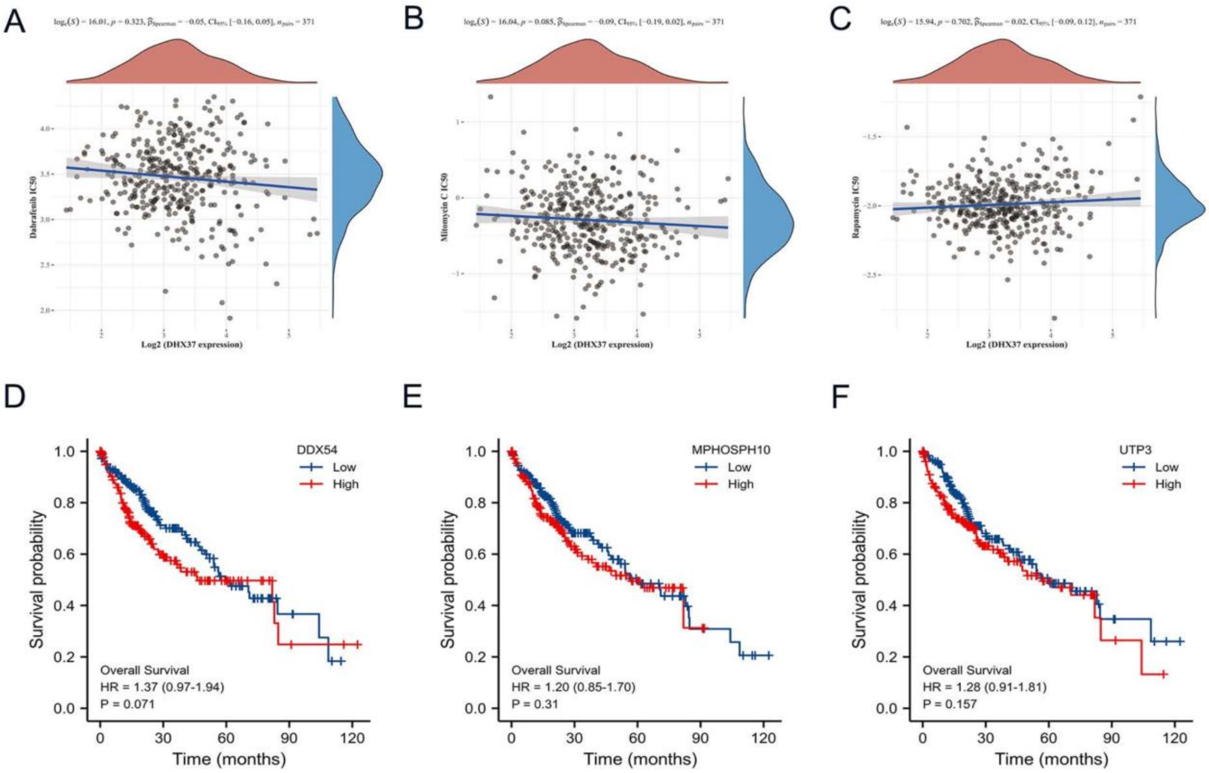

Additional file S1: Fig. S3 Chemotherapeutic drug sensitivity analysis and survival analysis. (A-C) Correlation of DHX37 expression with half-inhibitory concentration (IC50) of 3 chemotherapeutic drugs. (D-F) Survival analysis of DHX37-associated genes
